# Tumor‐agnostic detection of circulating tumor DNA in patients with advanced pancreatic cancer using targeted DNA methylation sequencing and cell‐free DNA fragmentomics

**DOI:** 10.1002/1878-0261.70116

**Published:** 2025-08-26

**Authors:** Morten Lapin, Kjersti Tjensvoll, Karin Hestnes Edland, Satu Oltedal, Herish Garresori, Bjørnar Gilje, Saga Ekedal, Trygve Eftestøl, Jan Terje Kvaløy, Filip Janku, Oddmund Nordgård

**Affiliations:** ^1^ Department of Hematology and Oncology Stavanger University Hospital Stavanger Norway; ^2^ European Liquid Biopsy Society (ELBS) Hamburg Germany; ^3^ Department of Electrical Engineering and Computer Science University of Stavanger Stavanger Norway; ^4^ Department of Mathematics and Physics University of Stavanger Stavanger Norway; ^5^ Department of Investigational Cancer Therapeutics The University of Texas MD Anderson Cancer Center Houston TX USA; ^6^ Department of Chemistry, Bioscience and Environmental Technology, Faculty of Science and Technology University of Stavanger Stavanger Norway

**Keywords:** cfDNA, cfDNA fragmentomics, ctDNA, DNA methylation, machine learning, pancreatic cancer

## Abstract

We investigated whether DNA methylation and cell‐free DNA (cfDNA) fragmentation patterns can improve circulating tumor DNA (ctDNA) detection in advanced pancreatic cancer. In a cohort of 33 patients, ctDNA detection was performed in a tumor‐agnostic fashion using DNA methylation, cfDNA fragment lengths, and 4‐mer 5′ end motifs. Machine learning models estimating ctDNA levels were built for each individual detection method and their combination. All models significantly differentiated ctDNA levels in patients from healthy individuals (*P* < 0.001). Using the highest estimated levels in healthy volunteers as cutoffs, ctDNA was detected in 79%, 67%, 67%, and 55% of patients using methylation, fragment length, end motifs, and the combined model, respectively. Univariable Cox regression showed that all ctDNA level estimates were associated with increased hazard ratios (HR, all *P* < 0.001) for progression‐free survival (PFS) and overall survival (OS). Multivariable Cox regression confirmed ctDNA levels as an independent predictor of PFS (HR = 1.9, *P* < 0.001) and OS (HR = 2.7, *P* < 0.001). Our findings suggest that machine learning models based on DNA methylation, cfDNA fragment lengths, and cfDNA end motifs can estimate ctDNA levels and predict clinical outcomes in advanced pancreatic cancer.

## Graphical abstract

We evaluated circulating tumor DNA (ctDNA) detection in advanced pancreatic cancer using DNA methylation, cell‐free DNA fragment lengths, and 5′ end motifs. Machine learning models were trained to estimate ctDNA levels from each feature and their combination. All models significantly distinguished patients from healthy individuals, and the ctDNA level estimations could be used to stratify patients according to survival.

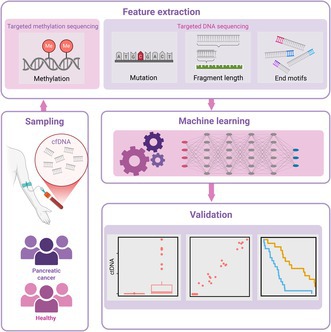

**Abbreviations**
cfDNA: cell‐free DNA
CHIP: clonal hematopoiesis of indeterminate potential
ctDNA: circulating tumor DNA
ECOG: Eastern Cooperative Oncology Group
HR: hazard ratio
MF: methylation fraction
OS: overall survival
PFS: progression‐free survival
REMARK: recommendations for tumor marker prognostic studies
RMSE: root mean square error
TCGA: The Cancer Genome Atlas
VAF: variant allele frequency

## 1 Introduction

Circulating tumor DNA (ctDNA) has shown great promise as a minimally invasive biomarker for patients with cancer [1, 2]. To detect ctDNA, it has been common to target somatic mutations using a tumor‐informed approach or hotspot mutations in common cancer genes [3]. Although sufficient for some clinical applications, the sensitivity of ctDNA detection based on somatic mutations may be inadequate for others [4]. Obstacles include a low rate of somatic mutations in many cancers, confounding factors such as clonal hematopoiesis of indeterminate potential (CHIP), and potential false negative results due to the limited shedding of cell‐free DNA (cfDNA) in early‐stage and some advanced‐stage cancers [4, 5, 6, 7]. These obstacles are prominent in pancreatic ductal adenocarcinoma (hereby referred to as pancreatic cancer), where ctDNA is undetectable in a fraction of patient samples even in advanced cancers, as demonstrated by us and others [8, 9, 10, 11, 12].

In recent years, advancements have been made in ctDNA detection methods that target other features of cfDNA, such as DNA methylation and characteristics of cfDNA fragmentation (cfDNA fragmentomics) [13, 14, 15, 16, 17, 18, 19, 20]. Unlike somatic mutations, which are often limited in number, DNA methylation alterations occur on a much larger scale and can also be specific to both tissue and tumor types [13, 18, 21, 22]. There is also evidence that DNA methylation can precede genetic changes during tumor formation [23, 24]. Furthermore, several features of cfDNA molecules, such as nucleosome positioning, fragment size, and fragment end motifs, have also been demonstrated to be highly discriminatory between healthy individuals and patients with cancer [14, 15, 16, 17, 20]. Aberrant DNA methylation and cfDNA fragmentation patterns could therefore potentially provide a complementary approach to ctDNA detection in patient samples where the detection of somatic mutations fails.

In this study, we investigated whether pancreatic cancer‐specific DNA methylation and cfDNA fragment features (fragment lengths and end motifs) could be used to improve the detection of ctDNA in patients with advanced pancreatic cancer. Individual machine learning models for different cfDNA features and a model combining detection methods were trained on cfDNA samples with known ctDNA levels from mutation‐based detection. Using the machine learning models, ctDNA levels were estimated in a group of 33 patients with advanced pancreatic cancer, including samples previously negative by mutation‐based detection. The prognostic value of ctDNA detection using the tumor‐agnostic methods and machine learning was evaluated by survival analysis.

## 2 Materials and methods

### 2.1 Patients and samples

**Fig. 1:**
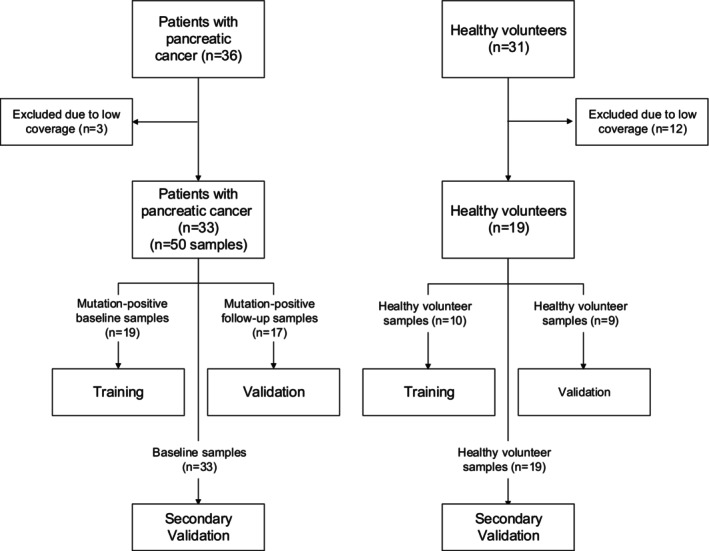
Overview of patient and volunteer samples used in the study. A subset of the patient samples (19 mutation‐positive baseline samples for training and 17 mutation‐positive follow‐up samples for validation) and all healthy volunteer samples (10 for training and 9 for validation) were used in the initial machine learning model development. Mutation‐negative baseline samples (*n* = 14) were excluded from training and validation to prevent false negatives from influencing the models. The secondary validation included the mutation‐negative baseline samples (*n* = 14), as well as the mutation‐positive baseline samples (*n* = 19) and healthy volunteer samples (*n* = 19) reused from the initial training and validation.

This study initially included 36 patients (Fig. 1) with advanced pancreatic cancer admitted to Stavanger University Hospital between October 2013 and October 2020. Three patients were excluded because of low sequencing coverage (< 50× deduplicated coverage). The coverage and dropout rate were influenced by cfDNA input levels. However, some samples were disproportionately affected by the bisulfite treatment, and this could not be explained by cfDNA preanalytical metrics. The remaining 33 patients (Table 1) had locally advanced (*n* = 6) or metastatic (*n* = 27) pancreatic cancer. Patients received first‐line treatment with gemcitabine (*n* = 1), gemcitabine plus nab‐paclitaxel (*n* = 20), or FOLFIRINOX (*n* = 12). Peripheral venous blood samples (9 mL K3EDTA tubes) were drawn before the initiation of chemotherapy (*n* = 33) and between chemotherapy cycles (*n* = 17). All blood samples were processed within 2 h of blood collection. The treatment response was defined by a standard disease evaluation of radiological images on the basis of the RECIST 1.1 criteria [25]. We also analyzed plasma from 31 healthy individuals older than 40 years with no prior or current cancer diagnosis as a negative control group (Fig. 1). A total of 12 plasma samples from healthy individuals were excluded because of low coverage. All patients and healthy controls provided written informed consent to participate in the study. The project was approved by the Regional Committee for Medical and Health Research Ethics (REK‐Vest 2011/475, REK‐Vest 2013/1743, REK‐Vest 27441). Experimental protocols followed the Declaration of Helsinki.

**Table 1:** Baseline patient characteristics.

Variable	Number (%)
Age	
Median (range)	66 (41–81)
Sex	
Female	11 (33)
Male	22 (67)
Primary tumor location	
Head	11 (33)
Body	6 (18)
Tail	7 (21)
Unknown or multiple	9 (27)
Tumor grade	
I	3 (9)
II	7 (21)
III	3 (9)
Unknown	20 (61)
Clinical T stage	
T1	0 (0)
T2	6 (18)
T3	8 (24)
T4	15 (45)
T×	4 (12)
Clinical M stage	
M0	6 (18)
M1	27 (82)
ECOG status	
0	5 (15)
1	20 (61)
2	8 (24)
First‐line treatment	
FOLFIRINOX	12 (36)
Nab‐paclitaxel/gemcitabine	20 (61)
Gemcitabine	1 (3)

### 2.2 Isolation of cfDNA from plasma

Blood samples were separated by density centrifugation using Lymphoprep™ (Serumwerk Bernburg, Bernburg, Germany) density gradient media, according to the manufacturer's instructions. We used 4 mL of plasma (diluted 1 : 1 with 0.9% NaCl for the density gradient separation protocol) to isolate the total cfDNA using the QIAamp MinElute ccfDNA Kit (Qiagen, Hilden, Germany), as described by the manufacturer. The cfDNA was eluted in 25 μL of ultraclean water (Qiagen) and stored at −80 °C until further analysis. cfDNA quantification was performed using a qubit 2.0 fluorometer and the dsDNA HS assay kit (Invitrogen, Carlsbad, CA, USA).

### 2.3 Methylation panel design

Hypermethylated CpG sites in multiple tumor types were identified in a previous study of data from The Cancer Genome Atlas (TCGA) [26]. From this dataset, we established a pancreatic cancer‐specific methylation panel. CpG sites were selected if they had more than 10% mean methylation in pancreatic adenocarcinoma tumor samples from TCGA. To increase specificity, we also required the CpG sites to have a maximum methylation of 2% in previously analyzed plasma cfDNA from 20 healthy volunteers [26]. Under these conditions, we designed hybridization capture probes targeting 450 CpG sites.

### 2.4 Library preparation and sequencing

Isolated cfDNA was bisulfite‐converted using the EZ DNA Methylation‐Lightning™ Kit (Zymo Research, Irvine, CA, USA) according to the manufacturer's instructions. The assay input was capped at 50 ng (range 2–50 ng). The bisulfite‐converted cfDNA was processed directly using the Accel‐NGS® Methyl‐Seq DNA library kit (Swift Biosciences, Ann Arbor, MI, USA) according to the manufacturer's recommendations with the following changes: The ratio of SPRI beads to the sample was 1.5× for the first two cleanups and 1.2× for the final cleanup, and KAPA HiFi HotStart ReadyMix (Roche, Pleasanton, CA, USA) was used to amplify the library. Libraries were amplified using 9–12 cycles, depending on the cfDNA input, producing ~ 1000 ng libraries for hybridization capture. Target capture was performed with the xGen hybridization capture of DNA libraries kit (Integrated DNA Technologies, IDT, Coralville, IA, USA) using biotinylated capture probes (120 bp), according to the manufacturer's instructions. The libraries were multiplexed 12‐plex; the hybridization time was increased to 16 h to allow efficient hybridization of the small panel (54 kb), and the captured DNA fragments were amplified using 13 cycles. The final libraries were sequenced on an Illumina NovaSeq 6000, using SP flow cells and 2 × 150 bp, producing ~ 4 m reads per sample. To increase base diversity, 10% PhiX was spiked into the libraries before sequencing.

### 2.5 Bioinformatics processing of bisulfite sequencing data

FASTQ files were generated using Illumina's bcl2fastq (v2.17.1.14). The reads were trimmed using trim galore (v0.6.1). To remove the 8 base polynucleotide tail at the 3′ end of each fragment introduced as part of the Accel‐NGS® Methyl‐Seq DNA Library Kit and to avoid low quality bases and biases in alignment, 10 and 15 bases were removed from '5 ends of Reads 1 and read 2, respectively. To retain symmetry for alignment, 15 and 10 bases were also removed from the '3 ends of Reads 1 and 2, respectively. Alignment and cytosine methylation calling were performed using bismark (v0.21.0) [27] running bowtie2 (v2.2.9), aligning read pairs to the bisulfite‐converted hg19 reference genome. The coverage of methylated and unmethylated CpGs 60 bp downstream and upstream of each capture probe CpG target was identified. Overlapping regions were merged, resulting in the merging of 36/450 (8%) targets. Furthermore, regions with < 0.2× median sequencing coverage (57; 12.7%) across all samples, and a region with extraordinarily high methylation in one of the healthy volunteers, were removed to produce 356 final target regions, the majority of which were in CpG islands (Data S2). Methylation in each target region was extracted from the aligned bam files using a custom script, and the number of methylated CpGs in each sequenced DNA fragment was calculated. To increase specificity, two methylated CpGs in each fragment were required for a fragment to be denoted as methylated. Thus, the methylation fractions (MFs) were calculated as the number of sequenced cfDNA fragments containing ≥ 2 methylated CpGs divided by the total number of cfDNA fragments mapped to each target region and presented as a percentage. Samples with a mean deduplicated coverage < 50× across the 356 target regions were excluded from further analysis. The median mean deduplicated coverage in the retained patient samples was 157 (range 89–495).

### 2.6 Bioinformatics processing of cfDNA fragmentation data

cfDNA fragment lengths and 4‐mer 5′ end motifs were extracted from off‐target reads obtained through targeted DNA sequencing of cfDNA in a previous study using the same patient samples as described above [9, 28]. The consensus sequences of completely sequenced off‐target cfDNA molecules were extracted using a combination of the tools sambamba (v0.8.0), samtools (v1.8), and the locally developed scripts tagxtractor (v1, https://github.com/oddmundn/TagXtractor) and sscscreator (v1, https://github.com/oddmundn/SSCScreator). The fragment length and end motif frequencies of the consensus cfDNA molecules were extracted from the aligned bam files using the locally developed Python script bamfrag (v2.1, https://github.com/oddmundn/BamFrag). Relative fragment length frequencies and end motif frequencies were calculated by dividing the read numbers by the total number of off‐target reads from each sample. The median total numbers of sequenced cfDNA fragments from the patient and healthy individual samples were 6.3E5 (range 1.3E5‐7.0E6) and 5.2E5 (range 2.2E5‐2.0E6), respectively.

### 2.7 Clustering and heatmap analysis

To visualize potential patterns in the measured cfDNA features, clustering and heatmap analyses were performed. cfDNA fragment length and end motif frequencies in all samples were first normalized against the mean frequencies in the samples from healthy volunteers and transformed by log2 transformation. Patient and control cfDNA samples were clustered according to transformed (cubic roots) MFs, fragment length frequencies (between 100 and 200 bp), and 4‐mer 5′ end motif frequencies by unsupervised hierarchical clustering, using the hclust function in r with a Euclidean distance measure. A complete linkage agglomeration method was used for the methylation data and ward.D method for fragment length frequencies and end motif frequencies. The order of individual methylated genomic regions was also determined by hierarchical clustering, using the same methods.

### 2.8 Machine learning‐based regression analysis

All the analyses were performed using the r package caret (v6.0‐94) in r (v4.4.0) using rstudio (v2023.12.1). Known ctDNA levels based on previous mutation‐based measurements [9, 28], transformed by cubic roots to increase normality, were modeled by multiple linear regression with regularization. MFs, also cubic root transformed to obtain a distribution closer to normal, cfDNA fragment length frequencies (between 50 and 200 bp), and end motif frequencies, were used as predictor variables. The models were trained on data from 19 baseline patient samples with known ctDNA content from previous mutation detection [9] and 10 randomly selected healthy volunteer samples (Fig. 1), with mutation‐based ctDNA content as the dependent variable. We performed the regression using elastic net regularization (combining the penalties of lasso and ridge regularization), utilizing the glmnet wrapper in the caret package and a leave‐one‐out cross‐validation method. Input values were centered and scaled using the ‘center’ and ‘scale’ methods of the preprocess function in the caret package. Individual models for DNA methylation, cfDNA fragment length, and end motifs were first built, followed by an ensemble model, combining the three individual models into a linear combination model. All the models were validated in a separate sample set with known ctDNA content (*n* = 17), obtained during follow‐up of the same patients and nine healthy volunteer samples, and analyzed by the same experimental methods (Data S1, Figs S1 and S2).

### 2.9 Other statistical analyses

The Mann–Whitney test was used to test for differences in continuous variables between independent groups. Progression‐free survival (PFS) was defined as the time between inclusion and progression according to the RECIST criteria or death due to any cause when the patient died before evidence of progression was obtained. Overall survival (OS) was defined as the time between inclusion and death due to any cause. The prognostic value of estimated ctDNA levels was assessed with Kaplan–Meier survival estimates, log‐rank tests, and univariable and multivariable Cox regression. The estimated and mutations‐based ctDNA levels were z‐score transformed before Cox regression was performed to enable direct comparisons of hazard ratios (HRs). Multivariable Cox regression was performed with backward selection of the variables, removing the least significant variable successively until all variables in the model had a *P*‐value < 0.1. The initial model included estimated ctDNA levels based on the combined data model, age, sex, primary tumor location, clinical M stage, Eastern Cooperative Oncology Group (ECOG) performance status, and first‐line treatment. The clinical T stage was omitted because of missing data. The proportional hazards assumption was checked with the cox.zph function in the survival package in r. All tests were two‐sided, and *P*‐values < 0.05 were considered statistically significant. This manuscript was prepared according to the REMARK guidelines (recommendations for tumor marker prognostic studies) [29].

## 3 Results

### 3.1 ctDNA measurements using targeted DNA methylation sequencing and cfDNA fragmentation patterns

**Fig. 2:**
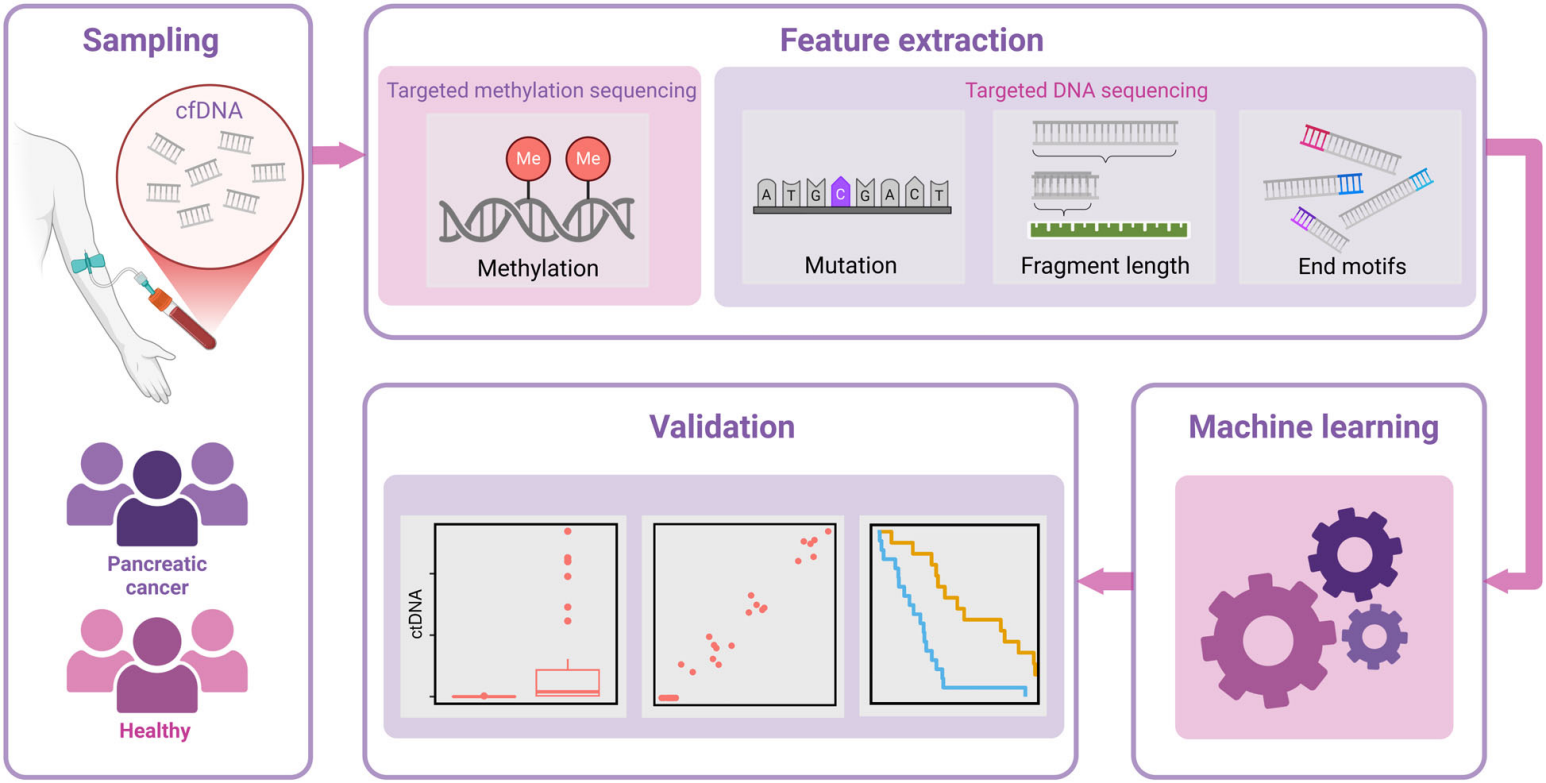
Overview of methods used in the study. Cell‐free DNA (cfDNA) was isolated from plasma collected from patients with advanced pancreatic cancer (*n* = 33) and healthy volunteers (*n* = 19). cfDNA features were extracted from targeted methylation sequencing performed in this study and from targeted DNA sequencing (fragment length and end motifs from off‐target reads) performed in a prior study [9], and used to generate machine learning models estimating ctDNA levels based on known ctDNA content from mutation‐based analyses. The machine learning models were validated by testing known vs. estimated ctDNA levels in patients and healthy volunteers, and by comparing estimated ctDNA levels with clinical outcomes. Made with BioRender (biorender.com).

We investigated whether we could detect ctDNA in a tumor‐agnostic fashion in plasma samples (*n* = 33) obtained from patients with advanced pancreatic cancer before initiation of chemotherapy. Plasma samples (*n* = 19) from healthy individuals constituted a ctDNA‐negative control group. cfDNA methylation analysis was performed using targeted DNA methylation sequencing, while cfDNA fragment lengths and 4‐mer 5′ end motifs were acquired from off‐target reads from previously performed targeted DNA sequencing of the same samples [9]. An overview of the included samples and methods used in this study can be found in Figs 1 and 2.

**Fig. 3:**
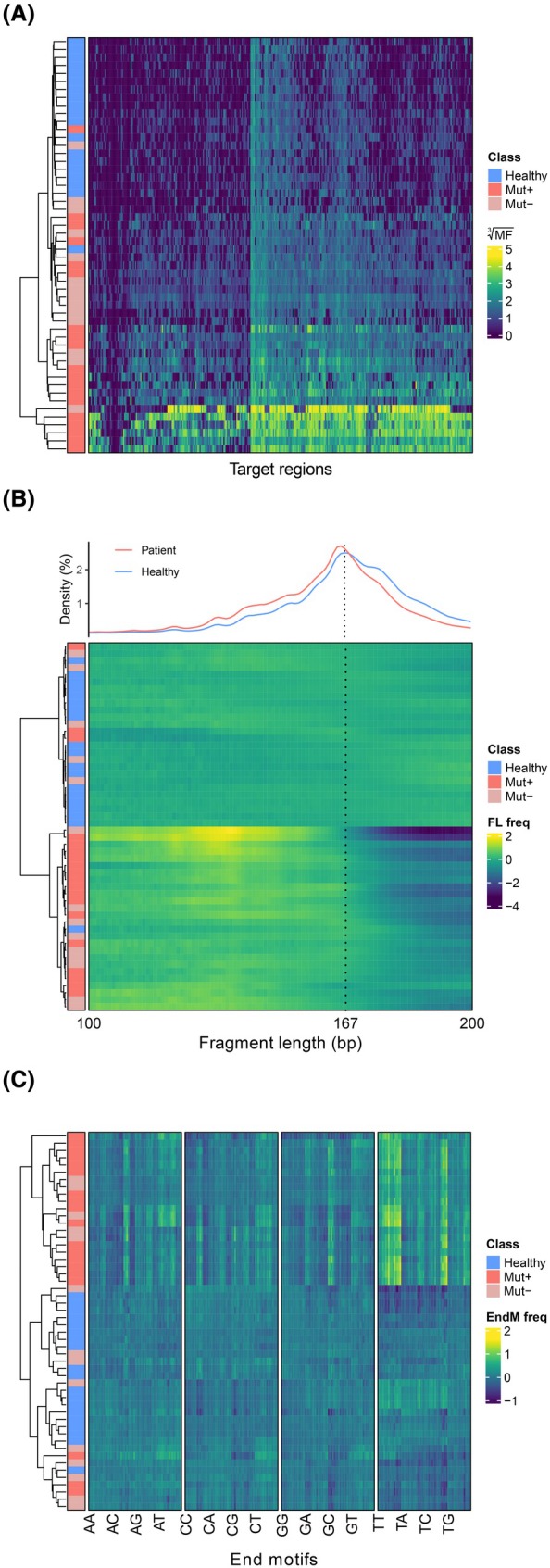
Tumor‐agnostic cell‐free DNA measurements. Hierarchical clustering and heatmaps from patients with advanced pancreatic cancer (*n* = 33) and healthy volunteers (*n* = 19) for; (A) cell‐free DNA (cfDNA) methylation fractions (MF); (B) relative cfDNA fragment length frequencies in plasma. The upper panel shows relative fragment length densities for fragment lengths between 100 and 200 base pairs, with the mode length for healthy volunteer samples (167 base pairs) indicated by a dotted vertical line. The lower panel shows a heatmap of relative fragment length frequencies (FL freq); (C) cfDNA end motif frequencies in plasma (EndM freq). All heatmaps show plasma samples in rows, ordered according to unsupervised clustering. Sample classes are shown with a colored annotation bar; blue denoting samples from healthy volunteers (Healthy); red denoting samples from patients with ctDNA detected by mutations (Mut+); pink denoting samples from patients without ctDNA by mutations (Mut−). Methylated target regions (A) are ordered by unsupervised clustering, fragment lengths (B) by size, and the 256 possible 4‐mer end motifs (C) in columns by base sequence. The color scale shows MFs transformed by computing cubic roots (A), and log2 ratios versus the mean of healthy control samples (B and C).

**Fig. 4:**
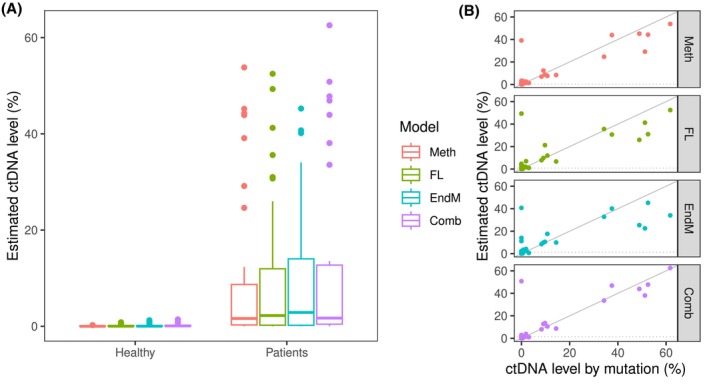
Estimated circulating tumor DNA levels in plasma from healthy volunteers and patients with pancreatic cancer. (A) Boxplot of circulating tumor DNA (ctDNA) levels in plasma obtained from healthy volunteers (*n* = 19) and patients (*n* = 33) with pancreatic cancer, estimated using machine learning models based on targeted methylation (Meth), cell‐free DNA fragment length (FL), end motifs (EndM) and a combined model (Comb). The boxplot shows median values, hinges (first and third quartiles), whiskers (smallest/largest values no further than 1.5 × inter‐quartile range from the hinges), and dots (‘outlier’ samples). (B) Scatterplot of estimated ctDNA levels in patient samples (*n* = 33) against previously measured ctDNA levels by mutation analysis. ctDNA levels are shown as percentages on a linear scale in both panels. The dotted horizontal lines indicate the cutoffs for positive ctDNA status for each method (=highest normal level), and the gray diagonal lines are unit lines.

For a subgroup of the patient samples, we observed increased methylation fractions for most target regions, as well as shorter cfDNA fragments and changes in end motif frequencies (Fig. 3A–C). We asked whether these findings could reflect the content of ctDNA in the samples and built machine learning‐based regression models for estimation of ctDNA levels using DNA methylation, cfDNA fragment length, and end motifs as predictor variables and mutation‐based ctDNA levels as the dependent variable. Modeling was first performed for each dataset individually followed by a combined model based on the individual models. The models were trained on mutation‐positive patient samples (*n* = 19) with a known variant allele frequency (VAF) and 10 randomly selected healthy volunteer samples. Validation of the models was performed in a separate set of mutation‐positive samples (*n* = 17) from the same patients, obtained at later time points, and nine healthy volunteer samples. The performance of the four regression models in the training and validation samples is shown in supplemental Figs S1 and S2 (Data S1), which demonstrate that the combined ctDNA data model had the best correlation to known ctDNA levels based on mutation detection. The methylation data contributed the most to the combined data model and, to a lesser extent, the fragment length data (Data S1, Fig. S3). Moreover, the ctDNA levels estimated by all the models were significantly greater in plasma from patients (*n* = 33) than in that from healthy individuals (*n* = 19) (*P* < 0.001; Fig. 4A). Interestingly, some patient samples that were negative for ctDNA by mutation analysis now had ctDNA detected using the tumor‐agnostic approaches (Fig. 4B). Using the highest estimated levels in plasma samples from healthy volunteers as cutoffs, ctDNA was detected in 26/33 (79%) patients by methylation, 22/33 (67%) by fragment length, 22/33 (67%) by end motifs, and 18/33 (55%) by the combined model, whereas 19/33 (58%) patients were detected using mutations. There was considerable overlap between the models, as 18/33 (55%) patients had estimated ctDNA levels above the cutoff estimated by all four models.

### 3.2 Prognostic value of ctDNA estimates

**Fig. 5:**
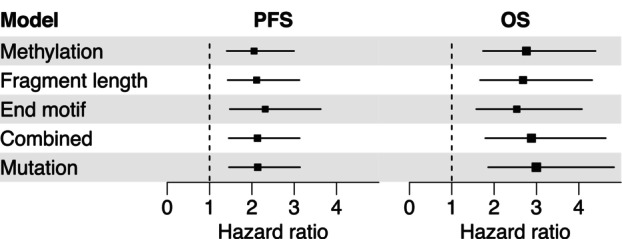
Univariable Cox regression analysis of estimated circulating tumor DNA levels. Hazard ratios for a one‐unit change in estimated circulating tumor DNA levels (transformed by cubic roots and divided by standard deviation, i.e., z‐score transformed), according to targeted methylation, cell‐free DNA fragment length, end motifs, the combined data model and measured mutation levels (variant allele frequency). Hazard ratios for progression‐free survival (PFS) and overall survival (OS) are shown as black squares and their 95% confidence intervals are indicated by the extending horizontal lines. The dimensions of the squares are proportional to the negative logarithm of the *P*‐values from the Cox modeling.

Survival analyses were performed to evaluate the clinical relevance of ctDNA levels estimated by our tumor‐agnostic approaches in comparison with mutation‐based ctDNA detection for the 33 baseline plasma samples. Univariable Cox regression demonstrated that all ctDNA level estimates were associated with increased HRs for both PFS and OS (Fig. 5). Only minor differences between the tumor‐agnostic estimates and mutation‐based measurements were observed. For OS, HR ranged from 2.54 to 2.88 for the tumor‐agnostic ctDNA level estimates compared with 3.0 for mutation‐based ctDNA levels. For PFS, the corresponding HRs ranged from 2.1 to 2.3 compared with 2.1.

**Fig. 6:**
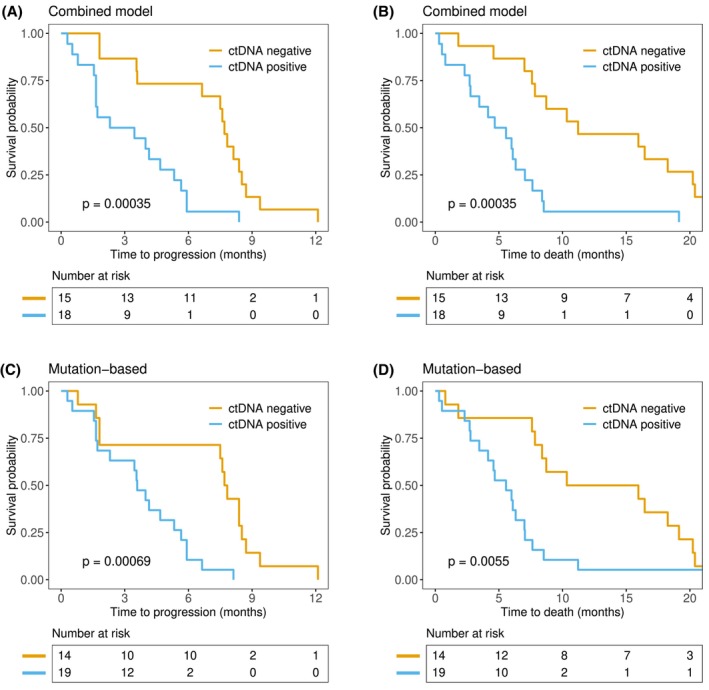
Kaplan–Meier estimates of progression‐free survival and overall survival. Patients were stratified according to circulating tumor DNA (ctDNA) detection by the combined tumor‐agnostic model (A and B) and mutation‐based ctDNA detection (C and D). For the combined model (A and B), a cutoff level based on measurements in plasma from healthy volunteers was used to stratify the patients. In the mutation model (C and D), the presence of mutations was used for stratification. The numbers of patients at risk at the indicated time points are shown below each plot. *P*‐values inside the plots are derived from log‐rank tests.

The Kaplan–Meier survival estimates confirmed the prognostic value of tumor‐agnostic ctDNA detection (combined model) in comparison with mutation‐based ctDNA detection (Fig. 6). The median PFS was 2.9 months (95% CI 1.6–5.7 months) for patients with ctDNA detected by the combined model and 7.7 months (95% CI 6.6–8.7 months) for those negative for ctDNA (Fig. 6A), whereas the median OS was 5.1 months (95% CI 2.8–7.6 months) and 11.2 months (95% CI 7.8–20 months), respectively (Fig. 6B). In comparison, the median survival times for mutation‐based ctDNA detection were 3.6 months (95% CI 2.3–5.9 months) vs. 7.8 months (95% CI 7.5–9.4 months) and 5.6 months (95% CI 4.1–7.6 months) vs. 13 months (95% CI 8.4–20 months) for PFS and OS, respectively (Fig. 6C,D). The Kaplan–Meier survival estimates for the individual models can be found in Fig. S4 (Data S1).

Owing to substantial correlation between the ctDNA models, only the ctDNA estimates based on the combined data model were included in the multivariable Cox regression, in addition to clinicopathological parameters (Data S1, Table S1). These analyses revealed that estimated ctDNA levels with the combined model, M stage, and first‐line therapy were independent predictors of PFS, whereas estimated ctDNA levels, ECOG performance status, clinical T stage, and age were independent predictors of OS (Table 2).

**Table 2:** Multiple Cox regression.

Variables	Progression‐free survival		Overall survival	
	Hazard ratio (95% CI)	*P*‐value	Hazard ratio (95% CI)	*P*‐value
Estimated ctDNA level^a^	2.1 (1.4–3.2)	**< 0.001**	4.0 (2.0–8.2)	**< 0.001**
Clinical M stage (M1 vs. M0)	3.9 (1.2–13)	**0.02**		
First‐line treatment (Nab‐pacl./gemcitabine vs. FOLFIRINOX)	2.6 (1.1–6.1)	**0.03**	2.7 (0.87–8.2)	0.08
ECOG status (2 vs. 0/1)			7.6 (1.7–34‐20)	**0.008**
Clinical T stage (T4 vs. T1/T2/T3)			0.36 (0.14–0.97)	**0.04**
Age (per year)			0.93 (0.87–0.98)	**0.01**

^a^ Per unit transformed and standardized level.

Significant *P*‐values are in bold.

## 4 Discussion

To increase the signal‐to‐background ratio of tumor to normal cfDNA in patients with cancer, multiple approaches have been introduced that leverage alternative features of cfDNA, such as DNA methylation and cfDNA fragmentomics (fragment length, end motifs, nucleosomal patterns, etc.), demonstrating promising results [13, 14, 15, 16, 17, 18, 19, 20]. Here, we investigated whether DNA methylation, cfDNA fragment lengths, and end motifs could be used to detect tumor‐derived DNA in plasma from patients with advanced pancreatic cancer and stratify patients according to prognosis. Machine learning models built using features from individual detection methods and a model combining both DNA methylation and cfDNA fragmentomics features successfully estimated ctDNA levels in patient plasma. The detection of ctDNA using these machine learning models was also demonstrated to be associated with decreased PFS and OS.

Methylation was the detection method that provided the most accurate model of known ctDNA content in this study and was also the main contributor to the combination model. An increasing number of studies have investigated cfDNA methylation in patients with pancreatic cancer (reviewed in [30]), especially as a marker for early cancer detection, although several recent studies have also investigated the prognostic potential of methylated cfDNA [31, 32]. Previous studies have been performed using both plasma/serum samples, single targets or panels, hypo‐ and hypermethylated genes, and several analysis methods. While some studies have demonstrated promising results, there seems to be a lack of consensus on the specific targets, methods used, and difficulties in reproducing results [30]. In this study, we designed a new, fairly large, methylation panel using data from TCGA and cfDNA data from healthy volunteers [26], with a focus on minimizing methylation levels in cfDNA from normal blood cells. As a consequence, many of the previously used methylation targets were not present in our panel. Although our methylation model for ctDNA had a similar or greater number of patients with ctDNA detected than did previous studies [30, 31, 32], it is possible that we excluded highly discriminatory targets by using a strict cutoff for methylation in healthy volunteer cfDNA. Furthermore, we also selected methylation targets using TCGA data at the group level and did not investigate whether certain targets were highly methylated in subsets of the TCGA samples, which could have increased ctDNA detection.

We observed that the amount of input DNA was lower for patient samples estimated to be ctDNA‐negative by DNA methylation than for samples that were ctDNA‐positive (*P* = 0.09, Mann–Whitney *U*‐test). We previously reported this also for mutation‐based detection [9], suggesting that increasing the amount of input DNA is highly important for increasing ctDNA detection. In this study, cfDNA was isolated from ~ 2 mL of plasma. While sufficient for most samples, increased plasma volumes would be required to reach the optimal input for all patient samples. An additional issue with low‐input samples for DNA methylation analysis is that these samples were, in general, most negatively affected by bisulfite treatment, further decreasing the sensitivity. As other methods, such as enzymatic conversion, are less prone to DNA damage [33], bisulfite treatment should perhaps not be the option of choice for DNA methylation analyses in the future, at least not when the amount of input DNA is scarce.

Previous results from our group have shown that cfDNA fragment length is significantly shorter in plasma from patients with pancreatic cancer than in that from healthy controls [34] and in ctDNA‐positive patients than in that from ctDNA‐negative patients [9]. Other studies have reported that shorter cfDNA fragment sizes are enriched in patient plasma [14] and that cfDNA fragment sizes differ between patient and healthy cfDNA and between cancer types in different regions of the genome [16]. It has also been demonstrated that cfDNA from patients with cancer, including those with pancreatic cancer, is preferentially enriched for certain 4‐mer 5′ end motifs [17, 35]. We therefore explored cfDNA fragment length patterns and end motifs in this study as a method to increase the sensitivity of ctDNA detection. Both the cfDNA fragment length, which we previously demonstrated to predict disease outcome in patients with advanced pancreatic cancer [34], and the end motifs provided prognostic information in this study. However, compared with DNA methylation detection, the use of cfDNA fragment lengths and end motifs provided less accurate machine learning models and contributed less to the combined model. Similar results were reported in the large Circulating Cell‐free Genome Atlas study, where DNA methylation outperformed other approaches for early cancer detection, including cfDNA fragment length and end motifs [19]. However, as we used off‐target reads with low sequencing depths to detect cfDNA fragmentation and end motifs, we did not have enough data to perform genome‐wide fragmentation pattern analyses. Ideally, low‐pass whole‐genome sequencing should be performed to fully capture the benefits of cfDNA fragment length and end motif analyses.

Our machine learning approach has several potential weaknesses. The first was that we had a very limited dataset available, containing many input features but few patient samples. To best overcome the risk of overfitting the models, we performed leave‐one‐out cross‐validation during model training and validated the models in a separate sample set obtained from the same patients at a later timepoint. Furthermore, we modeled our data using mutation‐based ctDNA levels as the dependent variable. This approach weighted features correlating with known mutation‐based ctDNA levels highest, thus potentially missing new information on DNA methylation and fragmentomics features that correlate with the presence of ctDNA independent of mutations. However, alternative machine learning approaches were tested (classification analyses comparing patients/healthy controls, regression using bagging, and machine learning‐based Cox regression), yielding similar results. Assuming that the mutation VAF can act as a surrogate for the ctDNA level is also a strong assumption, as these metrics are not necessarily correlated. Accordingly, a potential weakness of mutation‐based modeling was that we did not have any information about the homo‐ or heterozygosity of the mutations used to estimate VAF, which likely impacted the ctDNA estimates. However, to minimize errors in ctDNA level estimation, we did not include patient samples with known DNA amplifications in the genes included in the mutation sequencing panel.

The goal of this study was not only to determine whether DNA methylation and cfDNA fragmentomics could be used to detect ctDNA and provide prognostic information in patients with advanced pancreatic cancer but also to determine how these methods compare with mutation‐based ctDNA detection. Using the highest estimated ctDNA levels in plasma samples from healthy volunteers as cutoffs, we detected ctDNA in 67% of the samples using fragment lengths and end motifs and 55% and 79% for the combined model and DNA methylation, respectively. These numbers are greater than those of mutation‐based ctDNA detection in this study and greater for the DNA methylation model than for previous studies of mutation‐based ctDNA in advanced pancreatic cancer [9, 10, 11, 12]. However, the prognostic value of tumor‐agnostic ctDNA methods was not substantially different from that of the mutation‐based approach (Figs 5 and 6). Only one mutation‐negative patient with a high estimated ctDNA level (> 40%; Fig. 4B) had a very short PFS and OS (< 1 month), suggesting that the additional clinically relevant information provided by our tumor‐agnostic approaches is limited. Our models were built and tested using few patients and control samples, and the results should therefore be interpreted carefully. Testing our models in larger validation datasets would be necessary to confirm our findings.

## 5 Conclusions

In this study, we demonstrated that both DNA methylation and fragmentation patterns differ between patient and healthy volunteer plasma samples, and between patients with high and low levels of ctDNA. Machine learning models built on individual features (DNA methylation, cfDNA fragment lengths, and cfDNA end motifs) and a model combining all features could independently estimate ctDNA levels in patient samples; these estimations could be used to stratify patients according to PFS and OS. Comparisons of these models with mutation‐based detection of ctDNA led to a moderate increase in ctDNA detection, but it did not significantly improve stratification of patients according to survival.

## Conflict of interest

Filip Janku is an employee of Monte Rosa Therapeutics and has ownership interests in Monte Rosa Therapeutics (all unrelated to this work). All other authors declare no conflict of interest.

## Author contributions

ML, FJ, and ON conceptualized the study. ML, TE, JTK, FJ, and ON contributed to the methodology. ML, KT, and SO conducted the experiments. ML, KHE, HG, SE, BG, and ON curated the data. ML, KT, BG, TE, JTK, FJ, and ON interpreted the data. ON supervised the study. ML and ON drafted the manuscript. All the authors reviewed and commented on the manuscript and read and approved the final manuscript.

## Supporting information


**Fig. S1.** Correlation plots of estimated and measured circulating tumor DNA levels.
**Fig. S2.** Performance of circulating tumor DNA regression models in training and test set.
**Fig. S3.** Variable importance in the machine‐learning‐based regression models.
**Fig. S4.** Kaplan–Meier estimates of progression‐free and overall survival.
**Table S1.** Univariable Cox regression for clinicopathological parameters.

**Data S2.** Methylation panel CpG data.

## Data Availability

The data that support the findings of this study are not publicly available due to privacy restrictions. Datasets will be made available from the corresponding author upon reasonable request.
