# Supplementary material for: Tumor‐agnostic detection of circulating tumor DNA in patients with advanced pancreatic cancer using targeted DNA methylation sequencing and cell‐free DNA fragmentomics
Source: Mol Oncol. 2025 Aug 26;19(12):3535–47. doi: 10.1002/1878-0261.70116 (PMC12688167; doi:10.1002/1878-0261.70116)
Supplement: Supplementary file 1 — Fig. S1. Correlation plots of estimated and measured circulating tumor DNA levels. Fig. S2. Performance of circulating tumor DNA regression models in training and test set. Fig. S3. Variable importance in the machine‐learning‐based regression models. Fig. S4. Kaplan–Meier estimates of progression‐free and overall survival. Table S1. Univariable Cox regression for clinicopathological parameters. [file MOL2-19-3535-s001.docx]

**Tumor agnostic detection of circulating tumor DNA in patients with advanced pancreatic cancer using targeted DNA methylation sequencing and cell-free DNA fragmentomics**

Morten Lapin^1^, Kjersti Tjensvoll^1^, Karin Hestnes Edland^1^, Satu Oltedal^1^, Herish Garresori^1^, Bjørnar Gilje^1^, Saga Ekedal^1^, Trygve Eftestøl^2^, Jan Terje Kvaløy^3^, Filip Janku^4^,

and Oddmund Nordgård^1,5^

1 Department of Hematology and Oncology, Stavanger University Hospital, N–4068 Stavanger, Norway.

2 Department of Electrical Engineering and Computer Science, University of Stavanger, NO-4036 Stavanger, Norway.

3 Department of Mathematics and Physics, University of Stavanger, NO-4036 Stavanger, Norway.

4 Department of Investigational Cancer Therapeutics, The University of Texas MD Anderson Cancer Center, Houston, TX, USA

5 Department of Chemistry, Bioscience and Environmental Technology, Faculty of Science and Technology, University of Stavanger, Stavanger, Norway.

**SUPPLEMENTAL FILE 1**

**Supplemental Table 1: Univariable Cox regression for clinicopathological parameters**

|  |  | **Progression-free survival** | | **Overall survival** | |
| --- | --- | --- | --- | --- | --- |
| **Variable** | **n** | **Hazard ratio (95% CI)** | **P value** | **Hazard ratio (95% CI)** | **P value** |
| Age (per year) | 33 | 1.0 (1.0-1.1) | 0.25 | 1.0 (0.96-1.0) | 1.00 |
| Sex (male vs female) | 33 | 1.3 (0.64-2.7) | 0.46 | 1.0 (0.51-2.1) | 0.92 |
| Primary tumor location (body/tail vs head) | 22 | 2.4 (1.0-5.9) | 0.06 | 2.3 (1.0-5.7) | 0.07 |
| Clinical T stage (T4 vs T1/T2/T3) | 29 | 0.41 (0.18-0.93) | **0.03** | 0.46 (0.21-1.0) | **0.05** |
| Clinical M stage (M1 vs M0) | 33 | 2.7 (1.0-7.3) | **0.05** | 2.4 (0.89-6.2) | 0.09 |
| ECOG status (2 vs 0/1) | 33 | 3.6 (1.4-9.4) | **0.01** | 5.3 (1.9-15) | **0.001** |
| First-line treatment (Nab-pac+gemcitabine/gemcitabine vs FOLFIRINOX) | 33 | 1.2 (0.59-2.5) | 0.6 | 1.2 (0.57-2.4) | 0.7 |


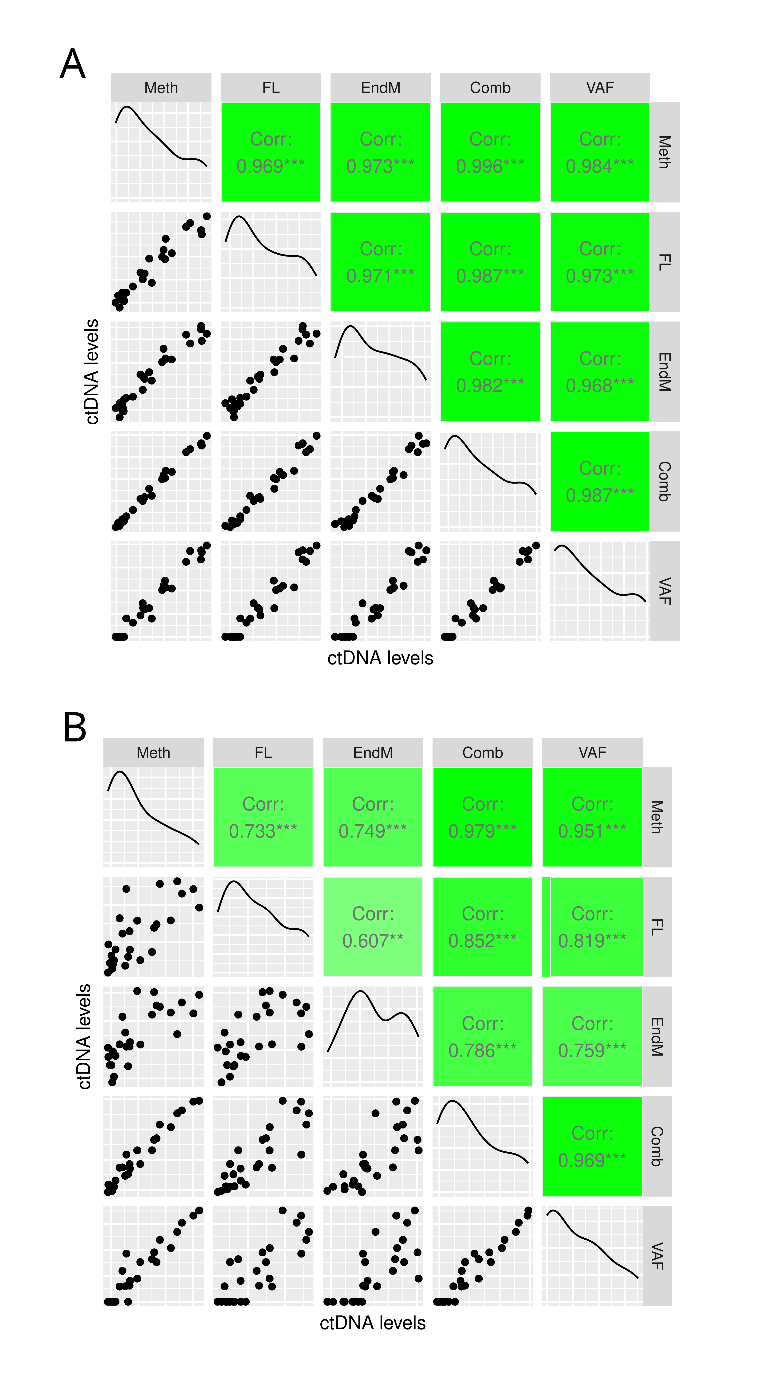


**Supplemental Figure S1: Correlation plots of estimated and measured circulating tumor DNA levels.** A) Scatterplots (lower left triangles) and correlation coefficients (upper right triangles) of comparisons between mutation-based (VAF) and tumor-agnostic estimated circulating tumor DNA (ctDNA) levels (cubic root transformed) in the training data set. B) Similar comparisons for the test set. Compared model estimates are based on targeted cell-free DNA (cfDNA) methylation sequencing (Meth), cfDNA fragment lengths (FL), cfDNA end motifs (EndM) and a combination of models (Comb). The P values of Pearson correlation tests are indicated in the upper right corners of the panels: *** = P value < 0.001, ** = P value < 0.01. The line plots in the diagonal positions display kernel density estimates for the ctDNA estimates represented by that specific row/column.


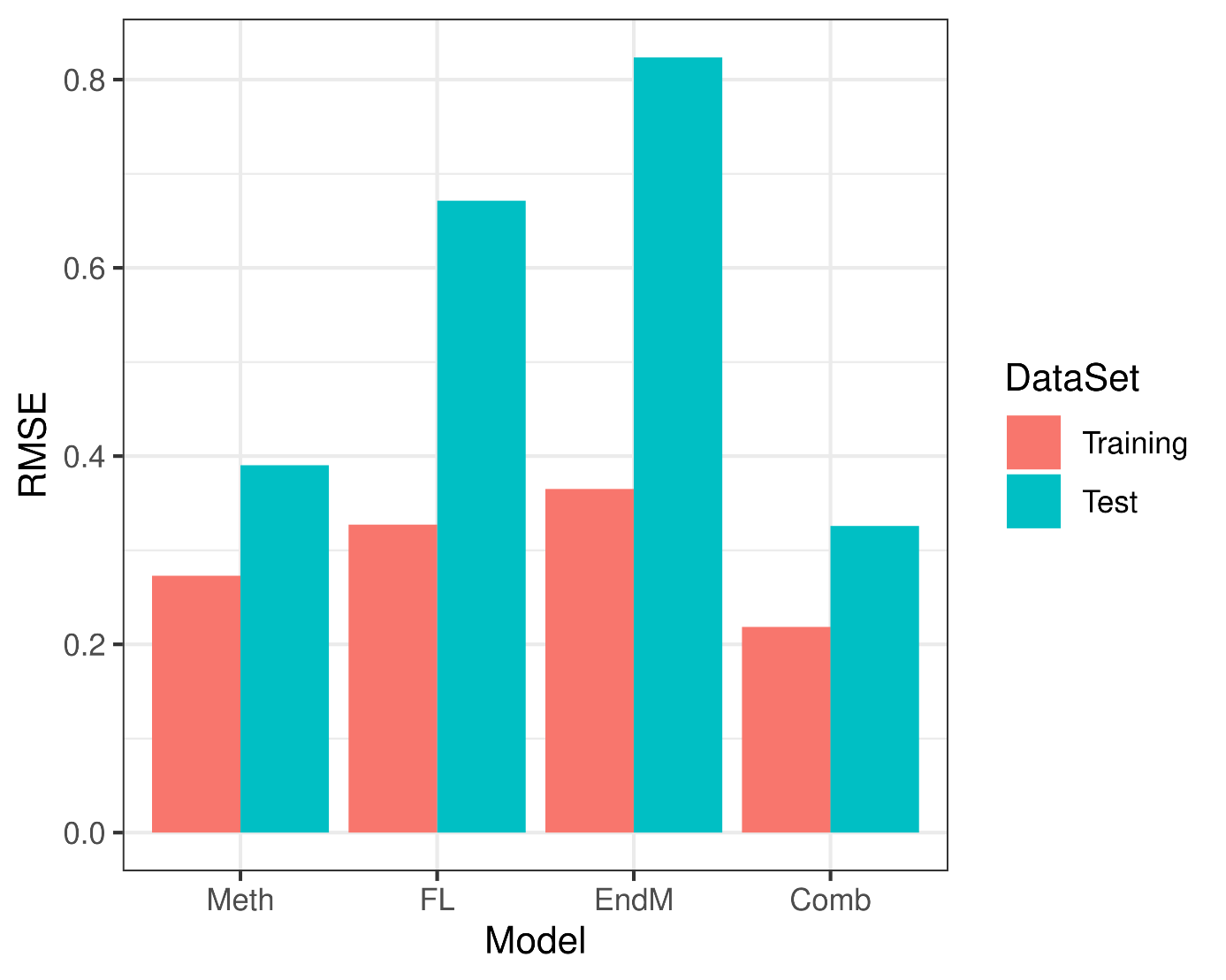


**Supplemental Figure S2:** **Performance of circulating tumor DNA regression models in training and test set.** Regression models for circulating tumor DNA level based on targeted cell-free DNA (cfDNA) methylation sequencing (Meth), cfDNA fragment lengths (FL), cfDNA end motifs (EndM) and a combination of models (Comb) were evaluated in the training and test data sets by computing the root mean squared error (RMSE) of the models.


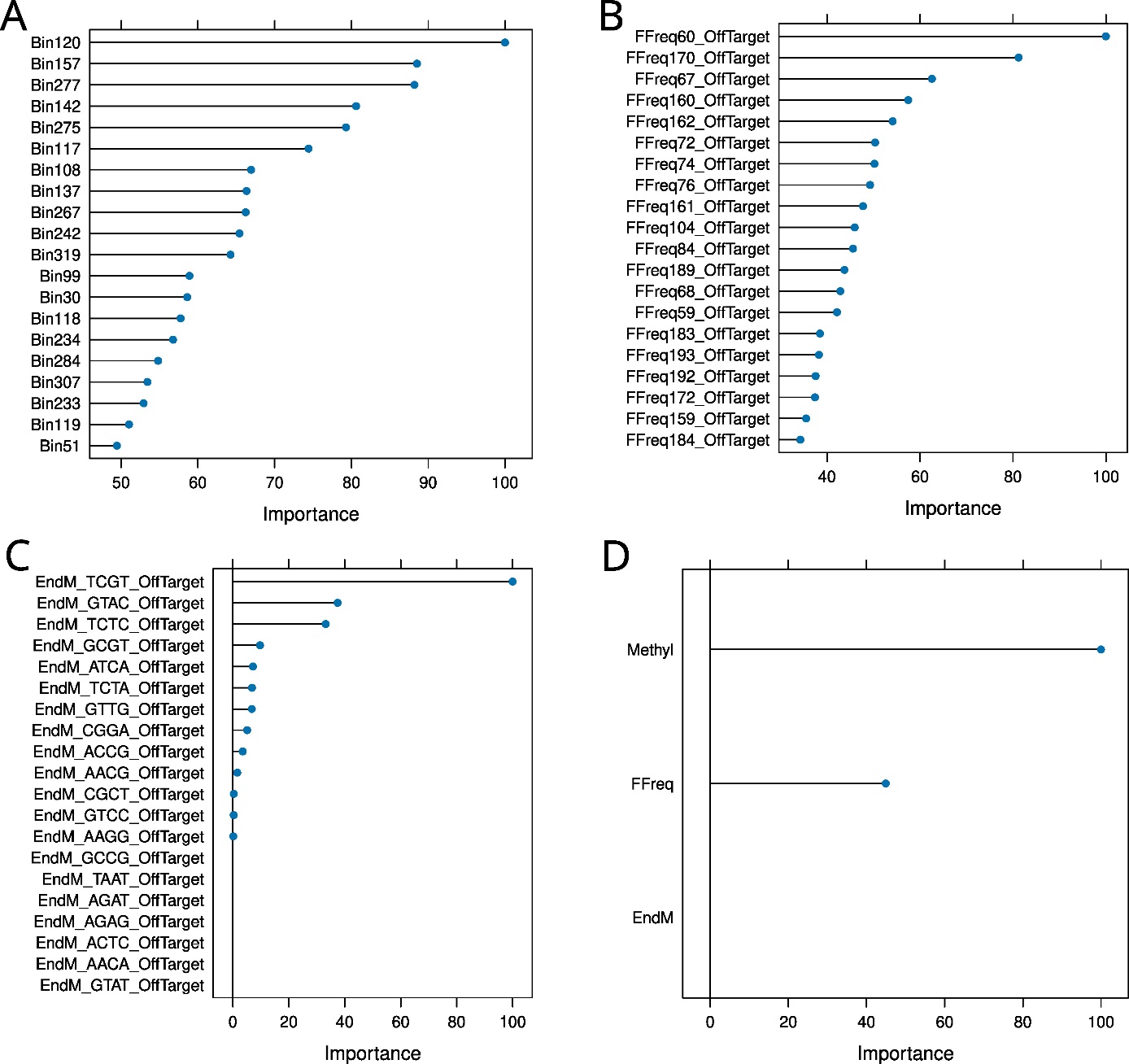


**Supplemental Figure S3: Variable importance in the machine-learning-based regression models.** Variable importance reported by the varImp function of caret/glmnet (as default adjusted to a scale from 0-100) for the methylation (A), fragment length (B), end motif (C) and combined models (D). A maximum of 20 input variables have been shown.


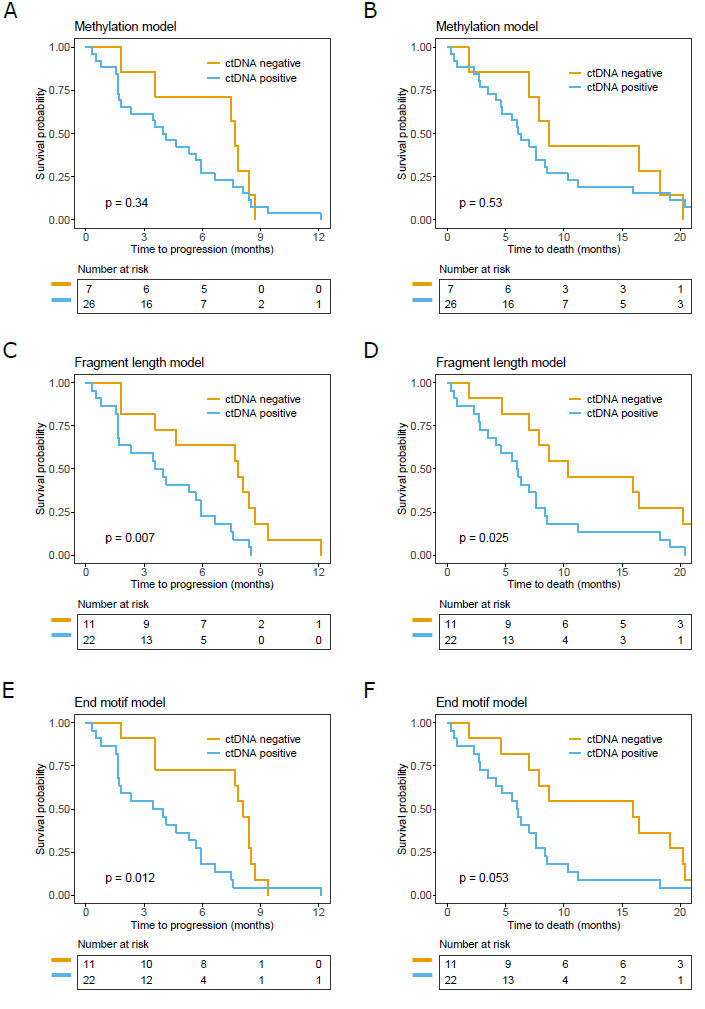


**Supplemental Figure S4: Kaplan-Meier estimates of progression-free and overall survival.** Patients were stratified according to circulating tumor DNA (ctDNA) detection by the DNA methylation model (A and B), Fragmenth length model (C and D), and End motif model (E and F). For all models, a cutoff level based on measurements in plasma from healthy volunteers was used to stratify the patients. Numbers of patients at risk at indicated time points are shown below each plot. P-values inside the plots are derived from log-rank tests.
